# Performance of Molecular Inversion Probe DR23K and Paragon MAD4HatTeR Amplicon Sequencing Panels for Detection of Plasmodium falciparum Mutations Associated with Antimalarial Drug Resistance

**DOI:** 10.21203/rs.3.rs-5743980/v1

**Published:** 2025-01-21

**Authors:** Thomas Katairo, Victor Asua, Bienvenu Nsengimaana, Stephen Tukwasibwe, Francis D Semakuba, Innocent Wiringilimaana, Shreeya Garg, Shahiid Kiyaga, Monica Mbabazi, Kisakye D Kabbale, Alisen Ayitewala, Samuel L Nsobya, Moses R Kamya, Issac Ssewanyana, Jeffrey A Bailey, Andrés Aranda-Díaz, Philip J Rosenthal, Bryan Greenhouse, Jessica Briggs, Melissa D Conrad

**Affiliations:** Infectious Diseases Research Collaboration; Infectious Diseases Research Collaboration; Infectious Diseases Research Collaboration; Infectious Diseases Research Collaboration; Infectious Diseases Research Collaboration; Infectious Diseases Research Collaboration; University of California, San Francisco; Infectious Diseases Research Collaboration; Infectious Diseases Research Collaboration; Infectious Diseases Research Collaboration; Uganda National Health Laboratory Services (UNHLS)/Central Public Health Laboratories (CPHL); Infectious Diseases Research Collaboration; Infectious Diseases Research Collaboration; Infectious Diseases Research Collaboration; Brown University; University of California, San Francisco; University of California, San Francisco; University of California, San Francisco; University of California, San Francisco; University of California, San Francisco

**Keywords:** Antimalarial drug resistance, MAD4HatTeR, DR23K, Molecular Inversion Probes, SNPs, Microhaplotypes, Sensitivity, Precision, Uganda

## Abstract

**Background:**

Molecular surveillance of drug-resistant *Plasmodium falciparum* is crucial for malaria control in endemic regions. Two targeted-resequencing tools, the Molecular Inversion Probe (MIP) drug resistance panel DR23K and the Multiplexed Amplicons for Drugs, Diagnostics, Diversity, and Differentiation using High-Throughput Targeted Resequencing (MAD^4^HatTeR) panel, are widely used to detect resistance genotypes. However, comparisons of their performance for genotyping drug resistance polymorphisms in malaria parasites and their comparative utility for other use cases is lacking.

**Methods:**

To compare the performance of DR23K and MAD^4^HatTeR in terms of sequencing depth, sensitivity to minor alleles, and precision, each platform was used to evaluate SNP alleles and microhaplotypes in double- and triple-strain mixtures of well-characterized laboratory parasites at densities of 10, 100, 1,000, and 10,000 parasites/μL. In addition, 67 Ugandan field samples collected in 2022 were genotyped using each platform to assess performance and concordance.

**Results:**

Across the four parasite densities, MAD^4^HatTeR exhibited superior sequencing depth (mean reads per locus: 144, 992, 1,153, and 1,300) compared to DR23K (mean unique molecular identifiers [UMIs] per locus: 1, 4, 49, and 364). For SNP detection, MAD^4^HatTeR achieved 100% sensitivity at 2% within-sample allele frequency (WSAF) at 1,000 and 10,000 parasites/μL, whereas DR23K achieved 100% sensitivity at 40% and 5% WSAF at these densities, respectively. Microhaplotype sensitivity was lower for both assays; MAD4HatTeR reached 69% sensitivity at 10 parasites/μL when WSAF was ≥ 10%, increasing to 100% sensitivity at 2% WSAF and 100 parasites/μL. DR23K had < 50% sensitivity at 10 and 100 parasites/μL. In field samples, which commonly contain polyclonal infections, high concordance was observed between the two methods for all SNPs (94%, 1,848/1,969) and polymorphic SNPs (88%, 898/1,019). All discrepancies were attributed to varied detection of minority alleles in mixed genotype infections.

**Conclusions:**

MAD^4^HatTeR demonstrated higher sensitivity than DR23K, particularly at low parasite densities. Both assays showed strong concordance for genotyping key resistance mutations in field samples, supporting their reliability. These findings suggest MAD^4^HatTeR as the preferred assay for low-density parasite studies and microhaplotype analysis, while DR23K may be appropriate for specific applications with high-parasite density samples, where detection of minority alleles is not prioritized, or when more comprehensive genome coverage is required.

## INTRODUCTION

Since 2000, significant progress has been made in reducing malaria transmission globally. Much of this progress can be attributed to mosquito control interventions and the utilization of efficacious drugs to treat and prevent malaria [1]. However, malaria remains a serious challenge in sub-Saharan Africa, causing extensive morbidity and mortality, especially in children [1]. There has been progress in reducing the burden of malaria in Africa, but this is threatened by the recent emergence of antimalarial and diagnostic resistance in *Plasmodium falciparum* and insecticide resistance in malaria vectors [1]. Overcoming these challenges will require improved molecular surveillance methods to inform policy decisions to maintain the efficacy of available drugs and diagnostics. Molecular surveillance is a crucial component of national malaria control programs, because it allows for scalable evaluation of *P. falciparum* genetic data to detect changes in parasite populations, [2] including insights into parasite transmission dynamics, relatedness, population connectivity, antimalarial drug resistance and vaccine antigen diversity [3].

For effective molecular surveillance of *P. falciparum*, accurate, sensitive, affordable, and scalable genotyping tools that can readily be adapted to changing surveillance needs are essential. Multiple targeted deep sequencing tools for malaria have been introduced [4–10], and two such tools are currently being utilized for high-throughput multi-locus genotyping of markers of drug resistance across sub-Saharan Africa, including Uganda, a country with emerging artemisinin partial resistance [11]. These tools are the molecular inversion probes (MIP)-based [12] drug resistance genotyping panel (DR23K) and a multiplex amplicon sequencing assay developed using Paragon Genomics CleanPlex technology (Multiplexed Amplicons for Drugs, Diagnostics, Diversity and Differentiation using High Throughput Targeted Resequencing, or MAD^4^HatTeR) [13]. Compared to Paragon CleanPlex technology, MIP technology allows for a high degree of multiplexing in a single reaction, and, due to its use of captures rather than amplification to target genomic regions of interest, can tolerate the inclusion of multiple probes in a single reaction with minimal impact on performance, allowing for multiplexing of different panels and addition of new targets with minimal optimization. In addition, MIPs do not require use of proprietary reagents, making the technology relatively inexpensive [12,14]. It also benefits from unique molecular identifiers (UMIs), which can be used to correct sequencing errors and quantify uncertainty in measures as they equate to the input template sequence. Paragon CleanPlex technology advantages include the capability to amplify targets from samples with low concentrations of template DNA with high sensitivity [13]. However, it relies on proprietary reagents. Despite the widespread usage of these two deep-sequencing platforms, data generated from the panels and analyzed using their respective bioinformatic pipelines have not been directly compared, limiting the ability of end users to make informed decisions for their specific use cases. To compare the sensitivity and precision of the two methods, we generated sequencing data for mixtures of well-characterized laboratory strains of varied parasitemia with known within-sample allele frequencies (WSAFs) and field isolates collected from patients presenting with symptomatic malaria in Uganda using published protocols. We then compared sequencing results for drug resistance loci targeted by both panels.

## MATERIALS AND METHODS

### Sample preparation and genotyping

#### Polyclonal laboratory controls

Controls were created using different proportions of well-characterized laboratory strains: 1) 99% U659 and 1% V1S, 2) 98% U659 and 2% V1S, 3) 95% U659 and 5% V1S, 4) 90% U659 and 10% V1S, 5) 60% U659 and 40% V1S, and 6) 60% D6, 30% FCR3 and 10% W2. All mixtures were serially diluted in erythrocytes to obtain parasite densities of 10–10,000 parasites/μL. 20 μL aliquots of each mixture were spotted on filter paper and stored at −20 °C until processing.

### Field Samples

Samples were collected from 67 patients aged 6 months or older presenting to Patongo Health Centre IV, Agago district with symptomatic uncomplicated falciparum malaria, diagnosed by rapid diagnostic test or blood smear, in 2022, as previously described [11]. Parasite density was determined by *varATS* quantitative PCR, as previously described [15]. The study was approved by the Makerere University School of Biomedical Sciences Research and Ethics Committee, the Uganda National Council for Science and Technology, and the University of California, San Francisco Committee on Human Research.

### DNA extraction

Genomic DNA was extracted from blood spots using Tween-20/Chelex 100 as previously described [16]. Briefly, 6 mm diameter discs were punched from DBS into 1.5 mL microcentrifuge tubes and incubated in 0.05% PBS-T (Tween 20 (Sigma Aldrich P9416) in 1X PBS (Corning 21–040-CV)) overnight at 4°C. Samples then underwent centrifugation at 13,000 rpm for 1 minute, the PBS-T was aspirated and replaced with 1 mL of 1X PBS, and the sample was incubated at 4°C for 30 minutes. Following 5 minutes of centrifugation at 13,000 rpm and 4°C, the supernatant was aspirated, and 150 μL of 7% Chelex 100 (Bio-Rad 1422822) in water was added. The sample was then incubated shaking at 95°C for 10 minutes. Following centrifugation at 13,000 rpm for 10 min, the supernatant containing extracted DNA was transferred to 1.5 mL sample storage tubes, avoiding transfer of any remaining Chelex beads. Extracted DNA was stored at −20°C.

### MADHatTeR amplicon panel and protocol

#### MAD^4^HatTeR amplicon panel and protocol

The primer pools used for the MAD^4^HatTeR amplicon panel were designed as previously described [13] and manufactured by Paragon Genomics. For this study, primer pools D1/R1.2 + R2 were used. D1 includes 165 high diversity targets and the *ldh* gene from *P. falciparum* and 4 other *Plasmodium* species (*P. vivax, P. malariae, P. ovale*, and *P. knowlesi*). R1.2 and R2 primer pools target 78 loci across 15 drug resistance genes, *hrp2/3* genes, 5 genes that cover potential vaccine targets including *csp*, and 4 non-falciparum *ldh* targets for species identification, as previously described [13]. The drug resistance targets include loci in *crt, mdr1, dhfr, dhps, kelch 13, coronin, exo, fd, arps 10, mdr2, pib7* and *ubp-1* genes; the assay also allows for evaluation of copy number of *hrp2/3, plasmepsin-2/3* and *mdr1* genes. A comparison of the regions of drug resistance genes targeted by the two panels is in **Supplemental Fig. 1** and the full protocol is online [17]. Two multiplexed PCRs were performed, one with primer pools D1 and R1.2 and the other with R2; two reactions are required to allow for tiling over genes of interest. After amplification, PCR products were pooled from the 2 reactions. The amplified DNA was then added to digestion reagents to degrade non-specific DNA products. Finally, indexing PCR was performed, wherein the cleaned DNA was amplified and indexed with 0.5 μM customized unique-dual indexing primers from Paragon Genomics. Pooling of the samples to create a sequencing library was performed as described in the **Supplemental Methods**.

### MIP panel and protocol

The DR23K MIP panel consists of 185 probes tiling across *P. falciparum* genes associated with drug resistance, including *crt, mdr1, dhfr, dhps, kelch 13, plasmepsin-2/3* **(Supplemental Table 1)**, allowing for genotyping of polymorphic sites that fall within targeted regions and assessment of copy number variation for all targeted genes. MIPs were designed using MIPTools software v 0.1.0 (https://github.com/bailey-lab/MIPTools) as previously described [12], and are a subset of probes utilized in drug resistance panels DR2 and DR3 [18]. MIP capture and library preparation were carried out as published previously [12,18]. Briefly, DNA was incubated in a mastermix containing MIP probes, dNTPs, ampligase, and Q5 polymerase, allowing for the hybridization of probes to targeted genomic regions, the targeted sequence was copied and ligated to fill gaps between the ends of the probes, generating a circularized product. In the following digestion step, exonucleases were added to digest linear DNA, leaving only circular probe-target sequence molecules, and PCR was conducted to amplify the circularized probe and add indexing barcodes. A detailed protocol is in **Supplemental Methods**.

### Library preparation and sequencing

Pooling strategies and library preparation methods are described in **Supplemental Methods**. Briefly, for both MAD^4^HatTeR and DR23K, sequencing libraries were composed of 96 pooled samples, including positive and negative controls, and sequenced on the Illumina Miseq platform using MiSeq v2 flow cells. MAD^4^HatTeR and DR23K libraries were sequenced independently on separate flow cells. For each sample, we allowed sequencing of 1) an initial library preparation 2) a single re-pool of the initial library for samples that did not reach 10 UMIs for at least 75% of the loci for DR23K or > 100 reads for at least 75% of the loci for MAD^4^HatTeR, 3) one repeat library preparation for failed reactions, and 4) a re-pool of samples that did not reach aforementioned coverage thresholds in the repeat library preparations.

### Data analysis

Illumina reads generated from MAD^4^HatTeR and DR23K sequencing libraries were analyzed using the MAD^4^HatTeR bioinformatic pipeline version 0.2.1 [13] and MIPTools version 0.4.0 (https://github.com/bailey-lab/MIPTools), respectively. Data from the MAD^4^HatTeR primer pool D1 were generated but not further analyzed in this study because D1 does not include drug resistance targets. Downstream analysis was performed using R version 4.2.2. For polyclonal controls, sequencing depth was calculated as the number of reads per target for MAD4HatTeR and as the number of UMIs recovered per target for DR23K. We further calculated per-sample coverage as the percentage of loci with ≥ 100 reads per sample for MAD4HatTeR and the percentage of loci with ≥ 5 UMI per sample for DR23K. Alleles were called using a cutoff of > 0.75% WSAF for MAD4HatTeR and 5 UMIs for DR23K. Microhaplotypes were identified as the unique sequence for the entire targeted region. Within-sample allele frequency (WSAF) was calculated as the proportion of reads called for that allele out of the total number of reads.

Laboratory assay and pipeline performance were evaluated by estimating sensitivity (ability to identify expected alleles; also known as recall) and precision (ability to identify only expected alleles) from polyclonal controls with different proportions of strains, using both SNPs and microhaplotypes as the allele of interest. Expected alleles (SNPs or microhaplotypes) were determined from whole genome sequencing data generated for monoclonal laboratory strains utilized in control mixtures. For MAD^4^HatTeR, microhaplotypes were evaluated following sequence correction and masking of low complexity (tandem repeats and homopolymers) as part of the bioinformatic pipeline [13]. For DR23KE, microhaplotypes were evaluated post sequence correction as implemented in the MIPTools pipeline, in which no masking is performed.

Observed (recovered) versus expected (known) WSAFs of the SNPs or microhaplotypes in the polyclonal controls was compared using linear regression. For field samples, concordance in genotype calls (whether a sample was determined to be pure wildtype, mixed genotype or pure mutant) was assessed. Comparisons of the abilities of the platforms to call SNPs from surveillance samples was performed using two-tailed Fisher exact tests evaluating differences in proportions of samples with missing genotype data for any of the drug resistance markers and proportions of loci across all samples that were not successfully genotyped.

### Role of the funding source

The funders of the study had no role in study design, data collection, data analysis, data interpretation, or writing of the report.

## RESULTS

### MAD^4^HatTeR provided higher sequencing depth than DR23K, particularly at low parasite densities

**Figure 1:**
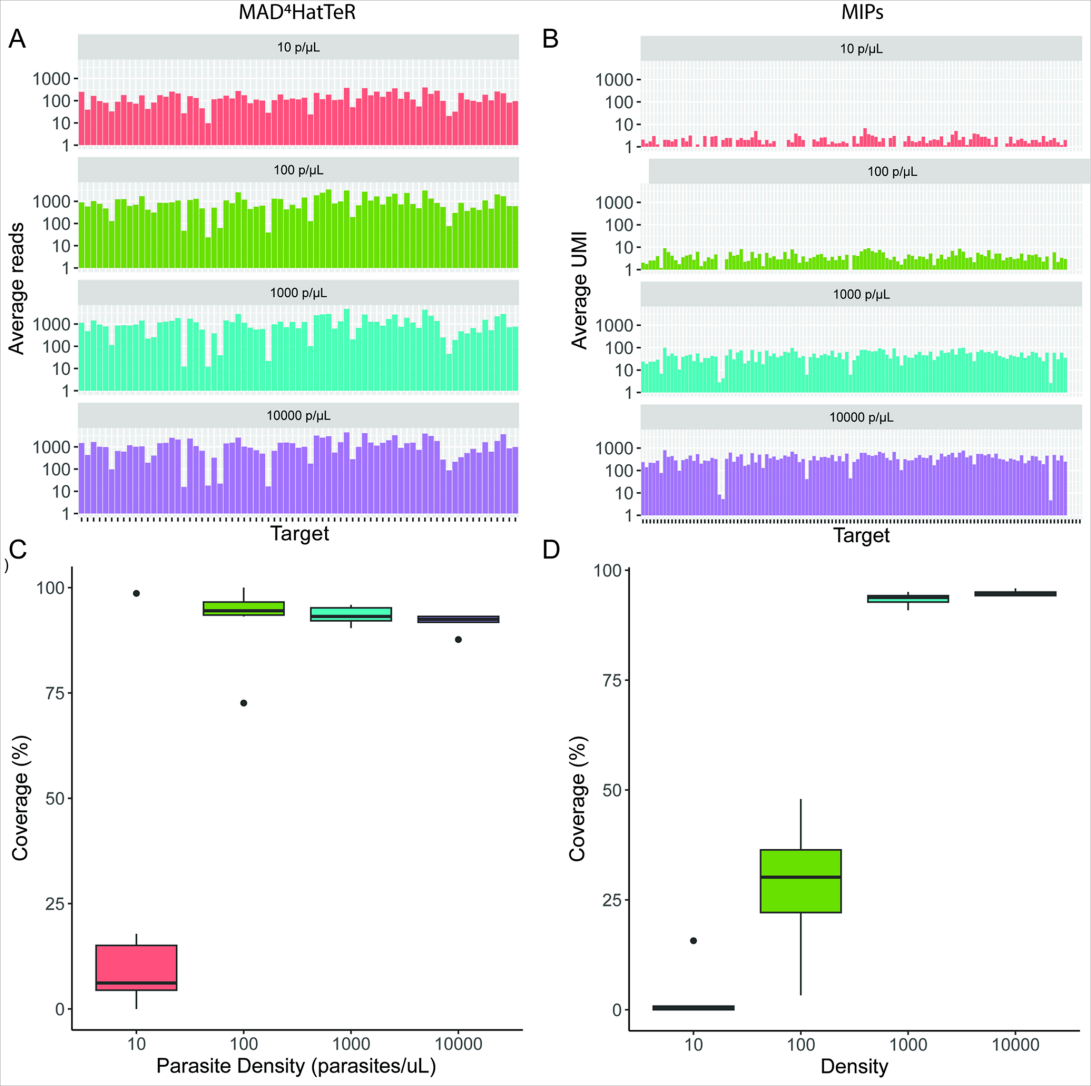
Sequencing depth achieved by MAD4HatTeR and DR23K stratified by parasite density, measured as A) average number of reads per locus for MAD4HatTeR and B) average UMI per MIP for DR23K. C) Median and IQR of percentage of loci with ≥ 100 reads per sample (coverage) for the MAD4HatTeR panel and D) median and IQR of percentage of loci with ≥ 5 UMI per sample (coverage) for DR23K. Six controls contributed data for each parasite density (10, 100, 1,000, and 10,000 parasites/μL).

Sequencing depth per target was compared for the two assays. For both assays, sequencing depth improved with increasing parasite density, as expected. Mean sequencing depth per locus for MAD^4^HatTeR was 144, 992, 1153, and 1300 reads for 10, 100, 1000, and 10,000 parasites/μL controls (Fig. 1A), while mean UMI coverage per MIP for DR23K was 1, 4, 49, and 364 UMIs, respectively (Fig. 1B). Sample-level coverage was compared by calculating the percentage of targeted loci for each sample with at least 100 reads for MAD^4^HatTeR or at least 5 UMI for DR23K. Using these criteria, MAD^4^HatTeR outperformed DR23K at 10 and 100 parasites/μL, with the greatest difference at 100 parasites/μL (median 94.5% for MAD^4^HatTeR and 30.2% for MIP, Fig. 1C and 1D). The incongruence between the high per-locus sequencing depth and low per-sample coverage at 10 parasites/μL for MAD^4^HatTeR was explained by one control at 10 parasites/μL that had significantly higher sequencing depth than the other controls at 10 parasites/μL.

### MAD^4^HatTeR was more sensitive than DR23K at detecting SNP alleles

**Figure 2:**
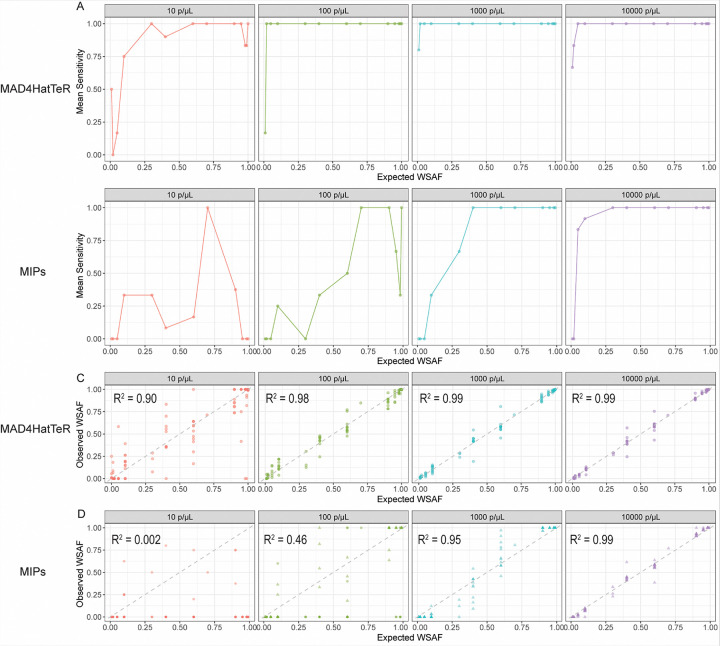
Mean sensitivity for SNP alleles across different WSAFs and parasite densities for A) MAD^4^HatTeR and B) DR23K. Observed versus expected SNP WSAFs for C) MAD^4^HatTeR and D) DR23K. R^2^ values were calculated using linear regression. Dotted line is y = x.

For both assays, sensitivity (ability to detect a SNP allele when it is present) improved as parasite density and WSAF increased. For MAD^4^HatTeR, sensitivity was 75% or greater when the WSAF was 10% or greater, and reached 100% when the WSAF was 2% at 100 parasites/μL (Fig. 2A). Compared to MAD^4^HatTeR, DR23K was less sensitive at all parasite densities (Fig. 2B); at 1,000 parasites/μL, 100% sensitivity required a WSAF of 40% and higher, and at 10,000 parasites/μL a WSAF of 5%. Across all parasite densities, MAD4HatTeR demonstrated superior sensitivity for detecting minority SNPs compared to DR23K, with the most pronounced differences observed at densities ≤ 1,000 parasites/μL.

Correlations between observed and expected SNP WSAFs were also assessed for both assays. For MAD4HatTeR, the R^2^ values were 0.90, 0.98, 0.99, and 0.99 at parasite densities of 10, 100, 1,000, and 10,000 parasites/μL, respectively (Fig. 2C). In contrast, for DR23K, the R^2^ values were 0.002, 0.46, 0.95, and 0.99 for the same parasite densities (Fig. 2D). Thus, MAD4HatTeR showed more accurate estimates of WSAFs than DR23K at densities ≤ 1,000 parasites/μL. Importantly, no false SNP genotyping calls (false positives) were observed with either method (Supplemental Figs. 2 and 3).

### MAD^4^HatTeR was more sensitive than MIP for detecting microhaplotype alleles

Sequencing of short, highly variable regions containing multiple SNPs (microhaplotypes) provides multiallelic information that improves evaluation of polyclonal infections. However, calling microhaplotype alleles is more challenging than calling SNPs, as these short amplicons may include regions of low complexity (tandem repeats and homopolymers) that are susceptible to PCR and sequencing errors. The MAD^4^HatTeR allele calling pipeline includes a masking step to exclude low complexity regions where sequencing errors are likely to occur, whereas the MIPTools pipeline does not. Therefore, the ability of each assay and pipeline to detect a true microhaplotype was assessed in addition to its ability to detect a true SNP.

**Figure 3:**
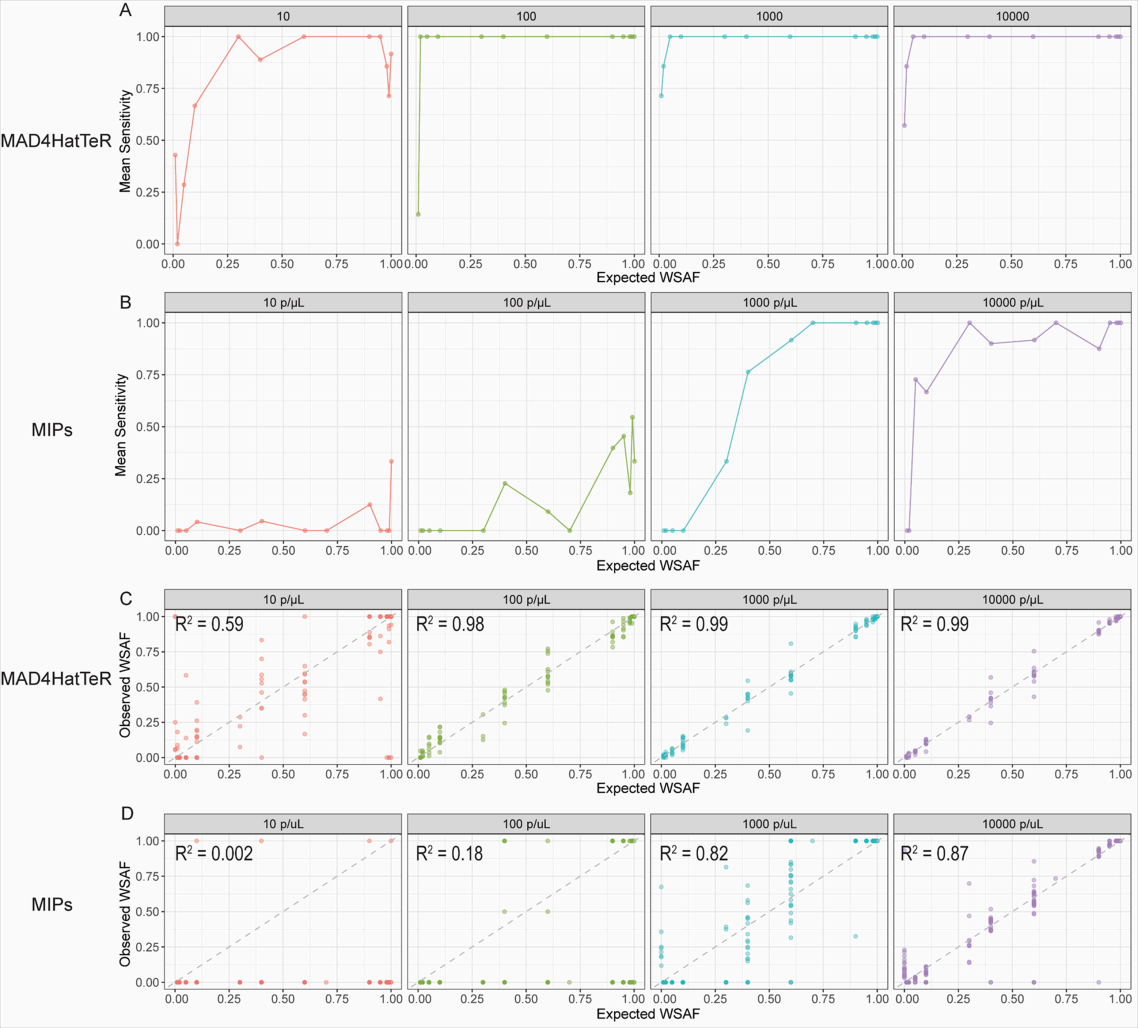
Mean sensitivity (recall) for microhaplotype alleles across different WSAFs and parasite densities for A) MAD^4^HatTeR and B) DR23K. Observed versus expected microhaplotype WSAFs for C) MAD^4^HatTeR and D) DR23K. R^2^ values were calculated using linear regression. Dotted line is y = x.

For both methods, sensitivity was lower for accurately detecting microhaplotypes than for SNPs, but the difference was larger for DR23K. For MAD^4^HatTeR at the lowest parasite density tested (10 parasites/μL), sensitivity was ≥ 69% when the true microhaplotype WSAF was ≥ 10% and reached 100% at a 2% microhaplotype WSAF at 100 parasites/μL (Fig. 3A). DR23K performed less well, especially at 10 and 100 parasites/μL, rising above 50% only at WSAF of 40% for 1,000 parasites/μL (Fig. 3B).

The correlation between the observed and expected microhaplotype WSAF for both methods was also assessed (Fig. 3C–D). As with SNP alleles, MAD^4^HatTeR consistently showed stronger correlation between expected and observed microhaplotype WSAFs across all parasite densities compared to DR23K. False positive microhaplotypes occurred with both methods (**Supplemental Figs. 3 and 4**). False positive microhaplotypes were called in the *mdr1* 86 locus in two of the double strain controls at 10 parasites/μL using the MAD^4^HatTeR assay. False positive microhaplotypes occurred with the DR23K assay in the 1,000 and 10,000 parasites/μL triple strain controls for *crt* MIPs #13, #18 and #22 and *dhps* MIP #1 (**Supplemental Fig. 5**). For DR23K, inspection of sequences suggested that all false positive haplotypes were the result of sequence variation in homopolymer tracts or 2 nucleotide motif-microsatellite repeats.

### High concordance between MAD^4^HatTeR and MIP for field samples

Concordance of genotypes called by the two methods was assessed across 33 SNPs of interest in *crt, mdr1, dhfr, dhps* and *kelch 13* in 67 samples from patients diagnosed with symptomatic malaria by malaria rapid diagnostic tests in Uganda. The median parasite density of these samples was 6023 parasites/μL (IQR: 602–81560). Beyond 2 samples missing data at > 85% of evaluated SNPs for both techniques (parasite densities of 3480 and 229 parasites/μL), only one additional sample missed one or more SNP calls by MAD^4^HatTeR (3/67, 4.5%). In contrast, 17 additional samples missed one or more SNP calls by DR23K (19/67, 28.4%, p = 0.0003), with lower parasite densities generally having poorer coverage (**Supplemental Fig. 6**).

Of the 33 SNPs considered, 17 were polymorphic, accounting for 1139 of the 2211 SNP genotypes evaluated (N loci x N samples). Consistent with results from control samples, more individual SNP calls were missed by DR23K than MAD^4^HatTeR (240/2211 vs. 56/2211, p < 0.0001). At loci where SNP calls were available from both methods (n = 1969/2211 for all SNPs and n = 1019/1139 for polymorphic SNPs), there was a high level of concordance between the two methods (1849/1969, 94% and 899/1019, 88%, respectively). There were no cases of discordant calls for pure mutant or pure wild type genotypes (Table 1). Consistent with the lower sensitivity of DR23K for minority alleles, discordant results were more frequently observed when MAD^4^HatTeR identified a mixed genotype and DR23K called a pure wild type (n = 51) or mutant (n = 40) allele, although there were also cases in which DR23K identified a mixed genotype and MAD^4^HatTeR called a wild type (n = 7) or mutant (n = 22) allele. Most discordant calls were in the *dhfr* and *dhps* genes. These were due to MAD^4^HatTeR detecting a mixed genotype in samples with low minority allele WASF when DR23K did not (**Supplemental Table 1**). Samples with lower parasite densities were more likely to have discordant genotype calls (**Supplemental Fig. 7**).

## DISCUSSION

Given the growing threat of antimalarial drug resistance in Africa, increasing deployment of molecular surveillance is driven by the desire of national malaria control programs (NMCPs) to evaluate geographically representative samples on a scale that allows for early detection of parasite polymorphisms associated with drug resistance. Molecular surveillance also has the potential to provide insights into diagnostic resistance, transmission dynamics, parasite connectivity, asymptomatic infections, therapeutic efficacy study results, and vaccine antigen diversity. It is important to select the most appropriate molecular tools to address NMCP goals, and choosing the right assay depends on the specific use case and the operational realities of a given setting. Important considerations include the assay’s reproducibility, ease of use, reagent availability, sensitivity, precision, scalability, and cost. More data from direct comparisons of these molecular tools allows better evaluation of the strengths and weaknesses of existing assays. Therefore, we compared the sensitivity and precision of the MAD^4^HatTeR and DR23K assays, methods currently utilized for surveillance in sub-Saharan Africa, for detection of SNPs and microhaplotypes at drug resistance loci, using laboratory controls with known strain mixtures and field samples.

MAD^4^HatTeR consistently provided more reliable coverage at low parasite densities, with higher sensitivity for detecting both SNPs and microhaplotypes in mixture controls. Sensitivity for detecting minority alleles and accuracy of estimation of WSAFs increased with parasite density for both assays, but MAD^4^HatTeR was more sensitive at lower parasite densities and lower WSAFs for both SNPs and microhaplotypes. While both methods demonstrated no SNP false positives, microhaplotype false positives occurred at low parasite densities with MAD^4^HatTeR and at higher parasite densities with DR23K. Despite these differences, concordance of SNP genotype calls was high in field samples, though DR23K missed more calls than MAD^4^HatTeR, particularly for mixed genotypes, underscoring its lower sensitivity.

MAD^4^HatTeR consistently demonstrated greater sensitivity than DR23K—particularly at lower parasite densities. MAD^4^HatTeR’s amplicon-based design provides high sensitivity without loss of precision, and its pipeline has been optimized for both SNP and microhaplotype analysis. These characteristics make it a useful tool for efficiently generating genotype data for a defined set of genomic targets across a wide range of parasite densities [19,20], especially when detection of minority alleles is a priority. However, tiling across genes requires two independent multiplexed PCR reactions, which increases template DNA requirements. Additionally, MAD^4^HatTeR relies on proprietary reagents from Paragon Genomics, making it reliant on a single supplier, and inclusion of new targets necessitates re-design of primer pools.

In contrast to MAD^4^HatTeR’s amplicon-based design, DR23K relies on MIP captures. This approach has advantages, such as not requiring proprietary reagents; allowing for tiling across genes in a single capture, which reduces the number of required reactions and the amount ofDNA template needed; and making the addition or removal of MIP targets from panels relatively straightforward. However, DR23K is notably less sensitive than MAD^4^HatTeR, a limitation inherent to the use of captures rather than primer-based amplification. In addition, the current MIPTools pipeline is not optimized for microhaplotype analysis. These characteristics may make DR23K less attractive for some uses. However, the ability to combine the DR23K panel with other, more expansive panels may be advantageous in locations where standard panels provide insufficient information for broad exploration of markers of emerging drug resistance.

The relative cost of assays must also be taken into consideration. Generalizable direct cost comparisons are difficult due to varied sequencing, reagent, and labor costs across countries, availability of negotiated discounts, and economies of scale. For example, MIP assays require upfront investment in probes and indexing barcodes, generally only available at minimum concentrations sufficient for hundreds of thousands of captures, which become more economical with large-scale projects. In contrast, MAD^4^HatTeR reagents are purchased by reaction, reducing upfront investment. MIP also requires fewer reactions to assay overlapping targets and is thus less labor intensive, which may make the assay more affordable. However, if good coverage is required for a large proportion of samples, the need for repeated captures and re-sequencing to achieve desired sequencing depth is likely to eliminate any savings, since the rate of re-sequencing is generally lower for the MAD^4^HatTeR assay. Thus, direct cost comparisons are likely to be lab and need-specific.

This study provides evidence that the MAD^4^HatTeR and MIP assays are reliable for surveillance of drug resistance, with high concordance in SNP calling in field samples. However, each assay has distinct advantages and limitations, and therefore is likely to be most useful in different studies and/or settings, emphasizing the need for multiple surveillance tools. For example, if the goal is to perform large scale genotyping with high parasite density samples (e.g. collected from patients with microscopicallydiagnosed, symptomatic malaria), MIP panels may be preferred over MAD^4^HatTeR due to cost efficiencies, with the knowledge that some data may be lost due to lower sensitivity. In addition, because tiling across genes is easier with MIPs (allowing for. multiplexing of targets an order of magnitude higher than Paragon technologies allow), this assay is better suited for identifying novel resistance markers or characterizing candidate markers. However, MAD^4^HatTeR has clear advantages in its ability to reliably provide accurate genotyping data and detect minority alleles in mixed samples, particularly at parasite densities < 1,000 parasites/μL, as is typical for samples from asymptomatic individuals with parasitemia [21,22]. In addition, MAD^4^HatTeR’s increased sensitivity would be the preferred choice for genotype correction to assign therapeutic efficacy study outcomes (with the addition of diversity markers), detecting mixed infections, and providing accurate microhaplotype data. As new assays are developed, it will be important to benchmark the sensitivity, precision, throughput, and cost of these assays versus existing tools.

In conclusion, compared to the DR23K panel, MAD^4^HatTeR demonstrated higher coverage and higher sensitivity for SNP detection, particularly at lower parasite densities, and outperformed DR23K in microhaplotype detection across all parasite densities. The methods showed high concordance in SNP calling, even when stratified for polymorphic SNPs in field samples, showing their reliability. As malaria genomics continues to expand, it is important to evaluate new tools. As every assay has strengths and weaknesses, we recommend that malaria molecular surveillance genomic assay selection be driven by specific use cases rather than chance or existing collaborations, as is often the case currently. We further emphasize that optimal malaria molecular surveillance will require the use of multiple, continuously evolving assays. Direct comparison of available assays will continue to be beneficial, helping to inform platform and panel selection, and guiding the innovation and optimization required to generate the next generation of surveillance tools.

## Figures and Tables

**Table 1:** Concordance of SNP genotype calls between MAD^4^HatTeR and DR23K for polymorphic SNPs in field samples

MAD^4^HatTeR Genotype Call					
DR23K Genotype Call		Wild Type	Mixed	Mutant	Missing
	Wild Type	575	51	0	0
	Mixed	7	75	22	0
	Mutant	0	40	250	0
	Missing	67	18	9	26

## Data Availability

Sequence data that support the findings of this study have been deposited in the NIH NLM Sequence Read Archive (SRA) with the primary accession code PRJNA1204991. Additional data is provided within the manuscript or supplementary information files.
